# Visceral Adipose Tissue Area as an Independent Risk Factor for Elevated Liver Enzyme in Nonalcoholic Fatty Liver Disease: Erratum

**DOI:** 10.1097/01.md.0000463680.47500.77

**Published:** 2015-03-20

In the article “Visceral Adipose Tissue Area as an Independent Risk Factor for Elevated Liver Enzyme in Nonalcoholic Fatty Liver Disease”,^1^ which appeared in Volume 94, Issue 9 of *Medicine*, Dr. Min Sun Kwak's name was spelled incorrectly in the byline. The article has since been corrected online.
